# Amentoflavone Exerts Anti-Neuroinflammatory Effects by Inhibiting TLR4/MyD88/NF-*κ*B and Activating Nrf2/HO-1 Pathway in Lipopolysaccharide-Induced BV2 Microglia

**DOI:** 10.1155/2022/5184721

**Published:** 2022-12-06

**Authors:** Shikuo Rong, Chunrong Yang, Feng Wang, Yiyang Wu, Kuishen Sun, Tao Sun, Zeyu Wu

**Affiliations:** ^1^Department of General Surgery, Guangdong Provincial People's Hospital, Guangdong Academy of Medical Sciences, Guangzhou, Guangdong 510080, China; ^2^Guangdong Cardiovascular Institute, Guangdong Provincial People's Hospital, Guangdong Academy of Medical Sciences, Guangzhou, Guangdong 510080, China; ^3^Ningxia Key Laboratory of Cerebrocranial Disease, Incubation Base of National Key Laboratory, Ningxia Medical University, Yinchuan, Ningxia 750004, China; ^4^Department of Gastroenterology, Hospital of Chengdu University of Traditional Chinese Medicine, Chengdu, Sichuan 610072, China; ^5^Department of Neurosurgery, The First Affiliated Hospital of Zhejiang University, Hangzhou, Zhejiang 310003, China

## Abstract

**Background:**

Amentoflavone, a natural biflavone, exerts anti-inflammation, antioxidation, and antiapoptosis effects on many diseases. However, the mechanism of amentoflavone on neuroinflammation-related diseases has not been comprehensively examined clearly.

**Methods:**

BV2 microglial cells were treated with amentoflavone (10 *μ*M), followed by lipopolysaccharide (LPS). Microglial activation and migration ability and the expression of proinflammatory cytokines and other signaling proteins were determined using immunohistochemistry, immunofluorescence, quantitative real-time polymerase chain reaction, Western blotting, enzyme-linked immunosorbent assay, and wound-healing assays.

**Results:**

Amentoflavone restored LPS-induced microglia activation, migration, and inflammation response which depends on regulating toll-like receptor 4 (TLR4)/myeloid differentiation factor 88 (MyD88)/nuclear factor kappa B (NF-*κ*B) pathway. In addition, amentoflavone also enhanced nuclear factor erythroid 2-related factor 2 (Nrf2)/heme oxygenase-1 (HO-1) levels in LPS-treated BV2 microglial cells.

**Conclusions:**

Amentoflavone ameliorated LPS-induced neuroinflammatory response and oxidative stress in BV2 microglia. These data provide new insight into the mechanism of amentoflavone in the treatment of neuroinflammation-related diseases. Therefore, amentoflavone may be a potential therapeutic option for neurological disorders.

## 1. Introduction

Neuroinflammation can trigger or aggravate several central nervous system (CNS) disorders, such as Alzheimer's disease [1], epilepsy [2], and Parkinson's disease [3]. Prolonged and repeated stimulation of inflammation can cause nervous system damage and neurodegeneration and lead to a progressive and permanent loss of neurons in specific regions of the brain [4]. Inflammation mainly occurs in microglia and affects the regulation of the immune system and the progression of neurological diseases [5]. Microglia activation exhibits a neuroprotective effect and plays a defensive role to counteract harmful acute damage. However, prolonged and repeated activation of microglia induces neuroinflammation and neurotoxic responses that can trigger or exacerbate nervous system damage and lead to the release of circulating cytokines, which eventually causes various CNS disorders [6, 7]. Therefore, anti-inflammatory therapies may be a promising strategy for treating neuroinflammation or neurodegeneration-related diseases.

The toll-like receptor (TLR) signaling pathway contains a family of receptors that recognize pathogen-associated molecular patterns (PAMPs) and can protect against invasive microorganisms that are detected from both the innate and adaptive immunities [8]. TLR4, which is mostly expressed in microglia, is one of the most important PAMPs in the TLR family [9]. TLR4 activation has been demonstrated to be responsible for chronic inflammation present in Alzheimer's disease [10], epilepsy [11, 12], Parkinson's disease [13], and other nervous system diseases [14]. TLR4 activation at the glial level can trigger a severe neuroinflammatory reaction. It is important to note that TLR4 can activate the downstream signaling pathways, which are strongly correlated with the development of CNS disorders, such as the myeloid differentiation primary response 88- (MyD88-) dependent and the NF-*κ*B signaling pathways; these pathways can trigger and aggravate the inflammatory response releasing various proinflammatory cytokines and mediators, such as interleukin-1*β* (IL-1*β*), tumor necrosis factor-*α* (TNF-*α*), cyclooxygenase-2, inducible nitric oxide synthase (iNOS), and the subsequent generation of nitric oxide (NO) [15]. Therefore, the TLR4/MyD88/NF-*κ*B pathway may be a promising target for treating neuroinflammation or neurodegeneration-related diseases.

It is well known that natural compounds with anti-inflammatory activity can be optimal candidates to develop effective therapeutic strategies. Amentoflavone is a natural biflavone with various biological properties [16]. Our previous experimental results have demonstrated that amentoflavone regulates NF-*κ*B p65, a key member of the TLR pathway or the oxidative stress pathway, to reduce the inflammatory response and exert a neuroprotective effect [17, 18]. However, the underlying mechanisms and functions of amentoflavone on neuroinflammation have not been fully investigated. In the present study, we analyzed the anti-inflammatory and neuroprotective effects of amentoflavone in lipopolysaccharide- (LPS-) induced BV2 microglia. To determine the mechanism of action of amentoflavone, the effects of amentoflavone were assessed on the TLR4/MyD88/NF-*κ*B pathway and the related inflammatory processes. To further investigate the neuroprotective effects of amentoflavone, the changes in the expression levels of the members of the nuclear factor erythroid 2-related factor 2 (Nrf2)/heme oxygenase-1 (HO-1) signaling pathway were also assessed.

## 2. Materials and Methods

### 2.1. Cell Lines and Culture Conditions

Mouse BV2 microglial cell line (National Infrastructure of Cell Line Resource, Beijing, China) was cultured in DMEM (Gibco, USA) containing 10% FBS (Corning, USA) at 37°C in 5% CO_2_. BV2 microglial cells were divided into four groups: control group, treated with 1% DMSO followed by PBS; LPS group, treated with LPS; LPS+AF group, treated with amentoflavone followed by LPS; and AF group, treated with amentoflavone alone.

### 2.2. Chemicals and Antibodies

Amentoflavone (THD256) was purchased from Ronghe Pharmaceutical Co., Ltd. (Shanghai, China). LPS (L2630) was purchased from Sigma-Aldrich (St Louis, MO, USA). Primary antibodies of TLR4 (ab13556), MyD88 (ab135693), iba-1 (ab178847), TNF-*α* (ab6671), iNOS (ab15323), and goat antirabbit IgG H&L (Alexa Fluor® 488) (ab150077) were purchased from Abcam (San Francisco, CA, USA). Primary antibodies of IL-1*β* (bs-0812R), p-I*κ*B (bs-2513R), NF-*κ*B p65 (bs-0465R), Keap1 (bs-3648R), Nrf2 (bs-1074R), PCNA (bs-0754R), and *β*-actin (bs-0061R) were purchased from Bioss Biotechnology Co., Ltd. (Woburn, MA). HO-1 (10701-1-AP) was purchased from ProteinTech Group (Wuhan, China). Peroxidase-conjugated goat antirabbit IgG (H+L) (ZB-2301) was purchased from Zhongshan Jinqiao Biotechnology Co., Ltd. (Beijing, China). Other general agents were commercially available.

### 2.3. Immunofluorescence Staining

BV2 microglial cells were treated with amentoflavone (10 *μΜ*) for 1 h, followed by treatment with LPS (1 *μ*g/ml) for 6 h. The cells were then fixed with 4% paraformaldehyde and then treated with 3% hydrogen peroxide for 10 min, respectively. After incubation with 3% BSA for 10 min, followed by anti-iba-1 (1 : 200) or IL-1*β* (1 : 500) overnight incubation at 4°C, cells were subsequently incubated with goat antirabbit IgG H&L (Alexa Fluor® 488) (1 : 500) for 1 h at RT. Cells were mounted with a sealer containing DAPI (ZLI-9557, ZSGB-BIO, Beijing, China), and images were captured with a Leica DM6 fluorescence microscope (Leica, Germany). The targeted areas were selected to quantitatively determine the integrated optical density (IOD) of fluorescence staining using ImageJ software (National Institutes of Health, USA) and to calculate the average optical density (AOD = integrated density/area).

### 2.4. MTT Assay

BV2 microglial cells were seeded into 96-well plates at a density of 4 × 10^3^ per well with amentoflavone at different concentrations (0, 0.01, 0.1, 1, 10, 25, 50, 75, and 100 *μ*M) in the absence of FBS. After 24 h incubation, cell viability was determined by MTT assay. In brief, MTT (5 mg/ml) was added into well plate for cell culture for 4 h at 37°C. The cell supernatant was then removed and 200 *μ*l of DMSO solution was added. Optical density was detected at 580 nM with a microplate reader.

### 2.5. Western Blot Analysis

BV2 microglial cells were treated with amentoflavone (10 *μΜ*) for 1 h, followed by treatment with LPS (1 *μ*g/ml) for 6 h. The total protein or nuclear protein from the BV2 microglial cells was prepared and extracted using the Membrane and Cytoplasmic Protein Extraction Kit (C510005, Sangon Biotech, Shanghai, China) or Nucleoprotein Extraction Kit (C500009, Sangon Biotech). Protein concentration was measured using the BCA Protein Assay Kit (KGP902, KeyGEN, Jiangsu, China). Equal amounts of protein (50 *μ*g per lane) were resolved on a 12% sodium dodecyl sulfate- (SDS-) polyacrylamide gel (SDS-PAGE) and then transferred onto 0.22 *μ*M polyvinylidene fluoride (PVDF) membrane (Millipore, USA). After blocking with 5% nonfat milk, rabbit anti-TLR4 (1 : 500), iba-1 (1 : 1000), MyD88 (1 : 2000), p-I*κ*B (1 : 500), I*κ*B (1 : 500), NF-*κ*B p65 (1 : 1000), IL-1*β* (1 : 500), TNF-*α* (1 : 1000), iNOS (1 : 1000), Nrf2 (1 : 500), Keap1 (1 : 500), HO-1 (1 : 1000), PCNA (1 : 500), and *β*-actin (1 : 2000) were incubated overnight at 4°C. Membranes were washed with PBST (PBS containing 1‰ Tween 20) and subsequently incubated with peroxidase-conjugated goat antirabbit IgG (H + L) (1 : 5000). Detection was performed with the automatic chemiluminescence gel imaging analysis system (Amersham Imager 600, General Electric Company, USA) and quantified via densitometry with ImageJ software. All experiments were performed in triplicate.

### 2.6. Measurement of Nitric Oxide (NO) Content

BV2 microglial cells were treated with amentoflavone (10 *μΜ*) for 1 h, followed by treatment with LPS (100 ng/ml) for 24 h. The supernatant was collected. The protein concentration was then determined with a BCA Protein Assay Kit (KeyGEN). The NO content was measured with the NO Assay Kit (Jiancheng, Nanjing, Jiangsu, China).

### 2.7. Enzyme-Linked Immunosorbent Assay (ELISA)

BV2 microglial cells were treated with amentoflavone (10 *μΜ*) for 1 h, followed by treatment with LPS (100 ng/ml) for 24 h. The supernatant of the cells was collected. IL-1*β* and TNF-*α* concentrations in tissue supernatants were measured with specific ELISA Kit (Cusabio Biotech, Wuhan, China) according to the manufacturer's protocol. The absorbance was measured at 540 nM in a microplate reader.

### 2.8. Measurement of Levels of GSH, MDA, and SOD

BV2 microglial cells were treated with amentoflavone (10 *μΜ*) or 0.5% DMSO for 1 h and then treated with LPS (100 ng/ml) for 24 h. Then, the measurement of GSH, MDA, and SOD was determined with assay kits (Jiancheng, Nanjing, China) in accordance with the manufacturer's instruction.

### 2.9. Wound-Healing Assay

Wound-healing assay was performed as described previously [19]. The BV2 cells were grown to 80% confluent in six-well culture plates and then wounded with 200 *μ*l pipette tips. The resultant unattached cells were removed and continuously cultured in serum-free culture medium for 24 h. The areas of cell movement during wound closure were observed and photographed. The gap in the area of closure was quantified with the ImageJ software. Percentage of migration (%) = (*A*1 − *A*0)/*A*1 × 100, where *A*1 represents the scratch area and *A*0 represents the remaining area of the wound at the metering point [20]. All experiments were performed in triplicate.

### 2.10. Statistical Analysis

Statistical analysis was performed using Prism 8 software. Results are presented as mean ± standard deviation (mean ± SD). One-way ANOVA was used for comparison of statistical differences among multiple groups, followed by the Dunnett or Tukey's significant difference test. All experiments were performed in triplicate.

## 3. Results

### 3.1. Amentoflavone Alleviates LPS-Induced Activation and Migration of Microglia in BV2 Microglial Cells

**Figure 1:**
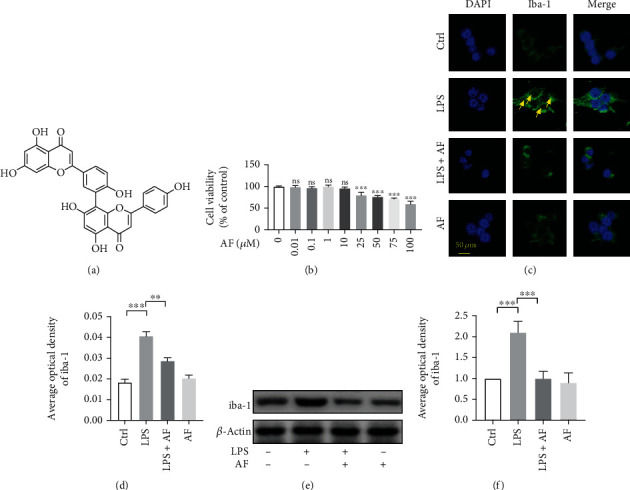
Effects of amentoflavone on LPS-induced activation of microglia in BV2 microglial cells. (a) Structure of amentoflavone. (b) Viability of BV2 microglial cells treated with amentoflavone in graded concentrations (0, 0.01, 0.1, 1, 10, 25, 50, 75, and 100 *μ*M) was measured by MTT assay (one-way ANOVA followed by Dunnett's test). (c) Representative images of immunofluorescence staining of iba-1 in BV2 microglial cells. (d) Semiquantitative analysis of the relative levels of iba-1 by densitometric analysis. (e) Representative immunoblots of iba-1 protein in BV2 microglial cells. (f) Semiquantitative analysis of the relative level of iba-1 by densitometric analysis. Error bars represent mean ± s.d. ^∗∗^ and ^∗∗∗^ represent *P* < 0.01 and *P* < 0.001, respectively. All experiments were performed in triplicate. LPS: lipopolysaccharide; AF: amentoflavone.

Initially, the appropriate concentration of amentoflavone was evaluated in BV2 cells. Our previous research studies demonstrated that the suitable experimental concentration of amentoflavone [17, 21] (Figures 1(a) and 1(b)) was 10 *μ*M, which was determined by the 3-(4,5-dimethylthiazol-2-yl)-2,5-diphenyl-2H-tetrazolium bromide assay (Figure 1(b)). Excessive activation of microglia exacerbates the development of inflammation, which in turn promotes the development of various neurological diseases. It is well known that iba-1 is a microglia-specific calcium-binding protein, which is commonly used as a marker of microglial activation [22]. Twenty-four hours following LPS stimulation, the expression of iba-1 was detected in BV2 cells (Figures 1(c)–1(f)). Compared with the control group, LPS-treated cells were characterized by large and densely stained cell bodies (Figure 1(c)). The fluorescence intensity of anti-iba-1 was significantly enhanced by LPS stimulation (*P* < 0.001, Figure 1(d)). In contrast to these observations, pretreatment with amentoflavone caused a reduction of this process and the cell morphology appeared similar to that of the control cells. However, amentoflavone did not affect the activation of microglia following single treatment of the cells with this compound (*P* > 0.05; Figures 1(c) and 1(d)). In addition, the effects of amentoflavone were examined on the expression levels of iba-1 in BV2 microglial cells using Western blotting. Amentoflavone could significantly inhibit LPS-induced iba-1 expression (*P* < 0.001); however, this compound did not affect iba-1 expression (*P* > 0.05; Figures 1(e) and 1(f)); these results are consistent with those derived from immunofluorescence staining.

**Figure 2:**
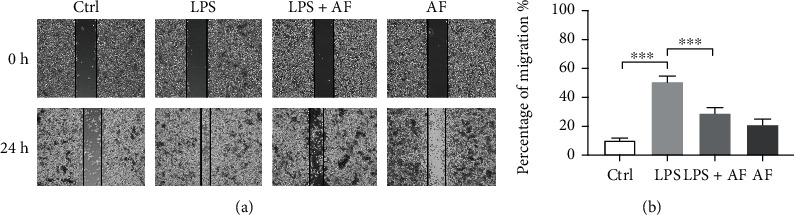
Effects of amentoflavone on LPS-induced migration of microglia in BV2 microglial cells. (a) Representative images of the wound-healing assays of BV2 microglial cells. (b) Analysis of migration indexes in different groups in BV2 microglial cells. Error bars represent mean ± s.d. ^∗∗∗^ represent *P* < 0.001, respectively. All experiments were performed in triplicate. LPS: lipopolysaccharide; AF: amentoflavone.

Microglial cell movement is associated with the induction of proinflammatory responses. Therefore, the present study examined whether amentoflavone could regulate LPS-induced BV2 microglial cell migration using wound-healing assays. The results indicated that treatment with LPS significantly increased BV2 microglial cell migration compared with that of the control group (*P* < 0.001). Amentoflavone significantly suppressed the migration induced by LPS (*P* < 0.001; Figures 2(a) and 2(b)). These data suggested that amentoflavone alleviated LPS-induced activation and migration of microglia in BV2 cells.

### 3.2. Amentoflavone Alleviates the LPS-Induced Inflammatory Response in BV2 Microglia Cells

**Figure 3:**
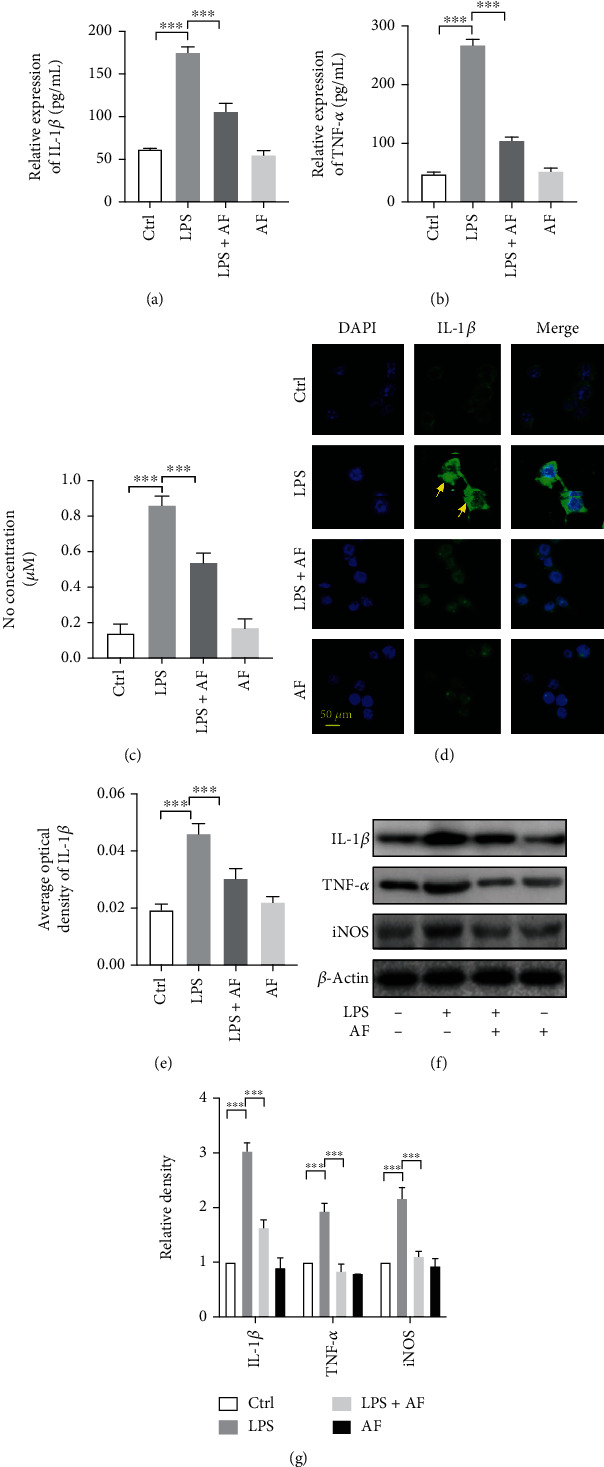
Effects of amentoflavone on LPS-induced inflammatory response in BV2 microglia cells. (a, b) Semiquantitative analysis of the relative levels of IL-1*β* and TNF-*α* using ELISA. (c) Semiquantitative analysis of the relative levels of NO. (d) Representative images of immunofluorescence staining of IL-1*β* in BV2 microglial cells. (e) Semiquantitative analysis of the relative levels of IL-1*β* in (d) by densitometric analysis. (f) Representative immunoblots of IL-1*β*, TNF-*α*, and iNOS protein in BV2 microglial cells. (g) Semiquantitative analysis of the relative level of IL-1*β*, TNF-*α*, and iNOS by densitometric analysis. Error bars represent mean ± s.d. ^∗∗∗^ represent *P* < 0.001, respectively. All experiments were performed in triplicate. LPS: lipopolysaccharide; AF: amentoflavone.

Subsequently, the effects of amentoflavone were assessed on neuroinflammation. The expression levels of the proinflammatory cytokines IL-1*β* and TNF-*α* were detected in BV2 microglia by ELISA and Western blotting, respectively. The results indicated that in BV2 microglia, the expression levels of the proinflammatory cytokines IL-1*β* and TNF-*α* in the LPS group were significantly higher than those in the control group (*P* < 0.001). However, amentoflavone significantly inhibited the expression levels of IL-1*β* and TNF-*α*, which were induced by LPS stimulation (*P* < 0.001). Moreover, single administration of amentoflavone to the cells did not affect the expression levels of these cytokines (*P* > 0.05; Figures 3(a), 3(b), 3(f), and 3(g)).

In addition, the effects of amentoflavone were investigated on iNOS expression and on the production of NO. The latter is a key inflammatory mediator of LPS-induced BV2 microglial inflammation. Similar to ELISA experiments, following 24 h of stimulation with LPS in BV2 microglia, NO production levels were detected. The results indicated that LPS significantly increased the expression of iNOS (*P* < 0.001, Figures 3(f) and 3(g)) and subsequently produced NO (*P* < 0.001, Figure 3(c)) compared with the control group. Amentoflavone significantly inhibited the expression levels of LPS-stimulated iNOS and the subsequent NO production (*P* < 0.001). However, single treatment of the cells with amentoflavone did not affect the expression of iNOS and subsequent NO production (*P* > 0.05; Figures 3(c), 3(f), and 3(g)). To further confirm these findings, the expression levels of IL-1*β* in BV2 microglia were examined by immunofluorescence analysis. The results were consistent with those of the ELISA and Western blot assays (Figures 3(d)–3(g)). These data suggested that amentoflavone alleviated the LPS-induced inflammatory response in BV2 microglial cells.

### 3.3. Amentoflavone Regulates the TLR4/MyD88/NF-*κ*B Axis to Alter LPS-Induced Proinflammatory Cytokine Levels

**Figure 4:**
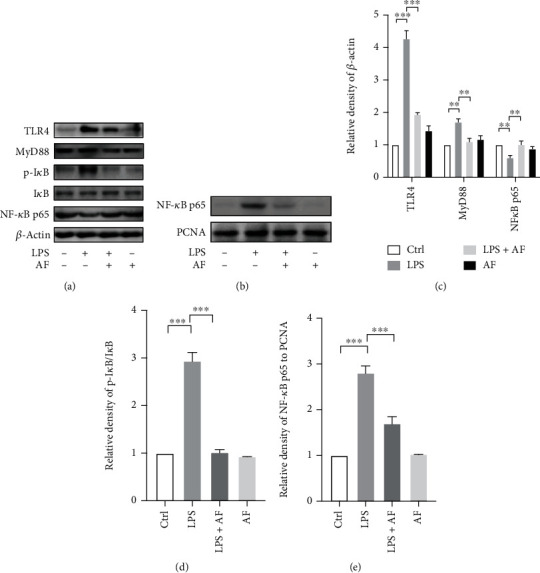
Effects of amentoflavone on regulating the TLR4/MyD88/NF-*κ*B axis to alter LPS-induced proinflammatory cytokine levels. (a) Representative immunoblots of TLR4, MyD88, p-I*κ*B, I*κ*B, and NF-*κ*B p65 proteins in the cytoplasm and cytomembrane in BV2 microglial cells. (b) Representative immunoblots of NF-*κ*B p65 proteins in the nucleus in BV2 microglial cells. (c, d) Semiquantitative analysis of the relative levels of TLR4, MyD88, p-I*κ*B, I*κ*B, and NF-*κ*B p65 in (a) by densitometric analysis. (e) Semiquantitative analysis of the relative levels of NF-*κ*B p65 in (b) by densitometric analysis. Error bars represent mean ± s.d. ^∗∗^ and ^∗∗∗^ represent *P* < 0.01 and *P* < 0.001, respectively. All experiments were performed in triplicate. LPS: lipopolysaccharide; AF: amentoflavone.

LPS stimulates and interacts with TLR4 to exert potent immune and inflammatory responses. NF-*κ*B is a ubiquitous and important nuclear transcription factor. It is a downstream effector of the TLR4 signaling pathway and mediates various inflammatory processes. Inactive NF-*κ*B is localized in the cytoplasm and binds to I*κ*B. Following phosphorylation of I*κ*B and its degradation, NF-*κ*B translocates into the nucleus and leads to the transcription of different proinflammatory genes, resulting in the production of inflammatory factors, such as IL-1*β* and TNF-*α* [23, 24]. However, amentoflavone can alleviate the LPS-induced inflammatory response in BV2 microglial cells. Therefore, it was hypothesized that amentoflavone regulates the TLR4/MyD88/NF-*κ*B axis to alter LPS-induced proinflammatory cytokine levels. Therefore, the protein levels of TLR4, MyD88, p-I*κ*B, I*κ*B, and NF-*κ*B p65 in BV2 microglial cells were detected by the Western blot analysis. Stimulation of the cells with LPS activated TLR4, MyD88, and I*κ*B (*P* < 0.01). Amentoflavone significantly inhibited LPS-induced expression of TLR4, MyD88, and I*κ*B (*P* < 0.01; Figures 4(a) and 4(c)) and promoted the transcription of NF-*κ*B p65 into the nucleus (*P* < 0.001; Figures 4(b) and 4(d)). These data suggest that amentoflavone inhibits the TLR4/MyD88/NF-*κ*B signaling pathway to alter LPS-induced proinflammatory cytokine levels.

### 3.4. Amentoflavone Enhances Nrf2/HO-1 Levels in LPS-Treated BV2 Microglial Cells

**Figure 5:**
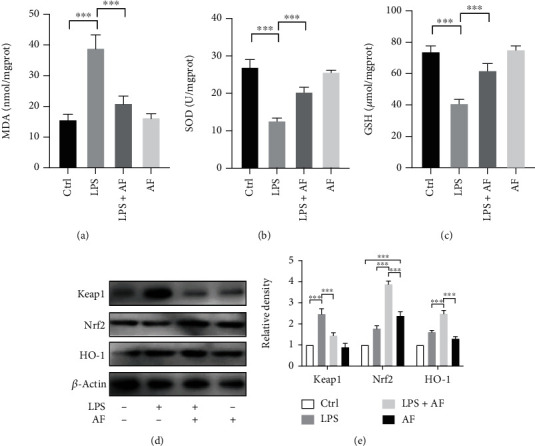
Effects of amentoflavone on the expression of Nrf2/HO-1 levels in LPS-treated BV2 microglial cells. (a–c) Semiquantitative analysis of the relative levels of MDA, GSH, and SOD. (d) Representative immunoblots of Keap1, Nrf2, and HO-1 proteins in BV2 microglial cells. (e) BV2 microglial cells were treated with amentoflavone (10 *μΜ*) or 0.5% DMSO for 1 h, followed by treatment with LPS (1 *μ*g/ml) or PBS for 6 h. Semiquantitative analysis of the relative levels of Keap1, Nrf2, and HO-1 by densitometric analysis. Error bars represent mean ± s.d. ^∗∗∗^ represent *P* < 0.001, respectively. All experiments were performed in triplicate. LPS: lipopolysaccharide; AF: amentoflavone.

Antioxidants play an important role in the prevention of neurological diseases. Therefore, the current study assessed the antioxidant effects of amentoflavone in LPS-treated BV2 microglia by assessing the activity levels and the content of malondialdehyde (MDA), glutathione (GSH), and superoxide dismutase (SOD). Similar to ELISA experiments, following 24 h of stimulation with LPS in BV2 microglia, the content of MDA, GSH, and SOD was detected. The results indicated that the MDA levels in the LPS group were significantly higher than those in the control group (*P* < 0.001; Figure 5(a)), while SOD and GSH levels were significantly lower than those in the control group (*P* < 0.001; Figures 5(b) and 5(c)). However, single treatment of the cells with amentoflavone did not affect the activity and expression levels of MDA, GSH, and SOD (*P* > 0.05; Figures 5(a)–5(c)). Furthermore, the present study investigated the effects of amentoflavone on the Nrf2/HO-1 signaling pathway in LPS-treated BV2 microglia. Subsequently, the protein expression levels of Kelch-like ECH-associated protein 1 (Keap1), Nrf2, and HO-1 were assessed by the Western blot analysis (Figures 5(d) and 5(e)). The results indicated that treatment with LPS significantly promoted the expression levels of Keap1 compared with those of the control group. However, pretreatment with amentoflavone significantly reduced LPS-induced Keap1 expression (*P* < 0.001; Figures 5(d) and 5(e)). Moreover, amentoflavone activated the expression levels of Nrf2 and HO-1 (*P* < 0.001; Figures 5(d) and 5(e)). LPS also caused a slight increase in the expression levels of Nrf2 and HO-1. However, the expression levels in the cells cotreated with LPS and amentoflavone were considerably higher than those in the cells treated with LPS and amentoflavone alone (*P* < 0.001; Figures 5(d) and 5(e)). These results suggest that amentoflavone activates Nrf2 and HO-1 and inhibits Keap1 expression, exerting antioxidant effects by regulating the Nrf2/HO-1 pathway in BV2 microglia.

## 4. Discussion

**Figure 6:**
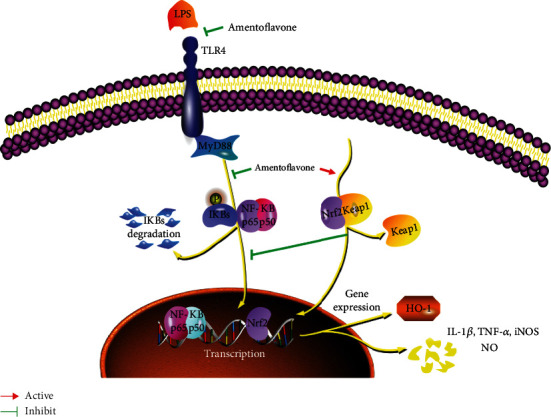
A schematic of a putative mechanism of amentoflavone in the progressive of neuroinflammation-related diseases. In this model, amentoflavone inhibits the TLR4/MyD88/NF-*κ*B and activating Nrf2/HO-1 pathway.

In the present study, amentoflavone alleviated LPS-stimulated microglial activation, migration, inflammatory response, and oxidative stress. These processes were dependent on the inhibition and activation of the TLR4/MyD88/NF-*κ*B and Nrf2/HO-1 pathways, respectively (Figure 6). Therefore, these findings indicated that amentoflavone may be a potential treatment option for various nervous system diseases.

Inflammation plays an important role in the pathophysiology of CNS-associated diseases. TLR4, a key receptor of innate immunity, is a critical driver of immune responses to bacterial infections and a critical link in various aberrant inflammatory responses. Following activation, TLR4 rapidly induces NF-*κ*B activation primarily through the MyD88-dependent signaling pathway in the inflammatory response, resulting in the release of proinflammatory cytokines, including IL-1*β* and TNF-*α*, to induce the development of CNS-associated diseases. The activation of TLR4 can clear amyloid beta (A*β*) accumulation at the initial stage; however, chronic long-term activation causes A*β* deposition in the brain, which causes or aggravates Alzheimer's disease [10]. The expression levels of high-mobility group box 1 (HMGB1) and TLR4 are increased in human epileptogenic tissues. Inhibition of HMGB1 and TLR4 retards seizure precipitation and decreases acute and chronic seizure recurrence, which are partly mediated by ifenprodil-sensitive N-methyl-d-aspartate receptors [12]. The HMGB1-TLR4 signaling pathway appears to be the most promising target for anticonvulsant and antiepileptic agents directed at inflammation [25, 26]. Following intracerebral hemorrhage, TLR4 is activated via the MyD88 signaling pathway causing brain oxidative injury and cognitive impairment [27]. TLR4-mediated inflammation may also be one of the key factors leading to neurodegeneration in Parkinson's disease [13]. Moreover, TLR4 mediates traumatic brain injury [28], cerebral cavernous malformations [29], spinal cord ischemia-reperfusion injury [30], and other neurological diseases [31, 32]. All these diseases occur mainly through TLR4-mediated inflammation, suggesting that TLR4 is an important therapeutic target. Disruption of LPS, which is an agonist of TLR4 and interacts with it during TLR4-mediated endocytosis, may inhibit the occurrence of inflammatory reactions, thereby alleviating or treating various neurological diseases [33]. Our previous data demonstrated that amentoflavone exerts a neuroprotective effect by inhibiting the NF-*κ*B-mediated inflammatory response, which is the key point for the function of TLR4 [18]. In the present study, the effect of amentoflavone on neuroinflammation was examined. LPS successfully induced a proinflammatory response in BV2 cells. However, these inflammatory responses were significantly inhibited by amentoflavone as determined by the mRNA and protein levels of the corresponding markers and mainly through suppressing the expression of the TLR4/MyD88/NF-*κ*B pathway markers, which is consistent with previous research results [34, 35]. These data indicated that amentoflavone could alleviate or treat various neuroinflammatory diseases mainly by regulating the TLR4/MyD88/NF-*κ*B signaling pathway.

In addition, oxidative stress also plays an important role in the development of neurological diseases. The activation of the transcription factor Nrf2 exerts neuroprotective effects and promotes neuronal survival; it also retards neuronal loss and disease progression during neurodegeneration and acute nerve damage [36, 37]. Nrf2 and its endogenous inhibitor, Keap1, evolutionarily conserved intracellular defense mechanism to counteract oxidative stress [38]. Nrf2 is involved in mitigating cellular damage induced by oxidative stress. The activation of Nrf2 modulates the transcription of the genes responsible for the antioxidant response, detoxification, and glutathione homeostasis and is mainly dependent on the cytoprotective enzyme HO-1 [37, 39]. Nrf2 interacts with both oxidative stress and inflammatory responses. The Nrf2/HO-1 signaling pathway is an indispensable signaling pathway for the antioxidative stress response, which also affects the occurrence and progression of neuroinflammatory diseases [40]. Amentoflavone exerts neuroprotective effects in Alzheimer's disease [41], epilepsy [17, 18], and Parkinson's disease [42]. It has been established that amentoflavone exhibits antioxidant and anti-inflammatory responses, which may act by inhibiting NF-*κ*B signaling [35]. In the present study, it was found that amentoflavone exerts antioxidant effects by activating Nrf2 in BV2 microglia. Activation of Nrf2 inhibits LPS-induced expression of proinflammatory cytokines, such as IL-1*β* and IL-6, and specifically inhibits NF-*κ*B-mediated inflammation-induced transcription [43]. In other words, the activation of Nrf2 inhibits the transcription of NF-*κ*B. It is concluded by the current findings and the previous data that amentoflavone can activate Nrf2, and it may inhibit LPS-induced NF-*κ*B activation via this process. This may be the potential link between the Nrf2/HO-1 and TLR4/MyD88/NF-*κ*B signaling pathways. However, further experimental, animal, and cellular studies should be performed to confirm this potential relationship.

In the present study, stimulation with LPS induced severe inflammatory responses and oxidative stress in BV2 microglia. However, amentoflavone ameliorated these effects by inhibiting the TLR4/MyD88/NF-*κ*B pathway and by activating the Nrf2/HO-1 pathway. These results suggest that amentoflavone may be a promising therapeutic agent for the treatment of neuroinflammation-related diseases.

## 5. Conclusions

Amentoflavone ameliorated LPS-induced neuroinflammatory response and oxidative stress in BV2 microglia. These data provide new insight into the mechanism of amentoflavone in the treatment of neuroinflammation-related diseases. Therefore, amentoflavone may be a potential therapeutic option for neurological disorders.

## Data Availability

All datasets generated for this study are included in the manuscript.

## Abbreviations

TLR4: Toll-like receptor 4
MyD88: Myeloid differentiation factor 88
NF-*κ*B: Nuclear factor kappa B
Nrf2: Nuclear factor erythroid 2-related factor-2
HO-1: Heme oxygenase-1
IL-1*β*: Interleukin-1*β*
TNF-*α*: Tumor necrosis factor-*α*
iNOS: Inducible nitric oxide synthase
NO: Nitric oxide
DMSO: Dimethyl sulfoxide
LPS: Lipopolysaccharide
CNS: Central nervous system
ELISA: Enzyme-linked immunosorbent assay
PBS: Phosphate buffer saline
RT: Room temperature.
